# Pingwei Powder alleviates high-fat diet-induced colonic inflammation by modulating microbial metabolites SCFAs

**DOI:** 10.3389/fcimb.2025.1628488

**Published:** 2025-09-16

**Authors:** Tangjuan Liu, Guosen Ou, Jialin Wu, Shiqi Wang, Hao Wang, Ziqi Wu, Yawen Jiang, Yaokang Chen, Huachong Xu, Li Deng, Xiaoyin Chen, Lu Xu

**Affiliations:** ^1^ School of Traditional Chinese Medicine, Jinan University, Guangzhou, China; ^2^ Department of Neurology, The First Affiliated Hospital of Jinan University, Guangzhou, China; ^3^ Foshan Clinical College, Guangzhou University of Chinese Medicine, Foshan, China; ^4^ Guangzhou Key Laboratory of Formula-Pattern of Traditional Chinese Medicine, School of Traditional Chinese Medicine, Jinan University, Guangzhou, China; ^5^ Guangdong Provincial Key Laboratory of Traditional Chinese Medicine Informatization, Jinan University, Guangzhou, China

**Keywords:** Pingwei Powder, gut microbiota, short chain fatty acids (SCFAs), autophagy, herbal medicine

## Abstract

**Background:**

Pingwei Powder (PWP), a renowned traditional Chinese medicine (TCM) formula, it has demonstrated excellent therapeutic effects in ulcerative colitis (UC), yet its underlying pharmacological mechanisms remain unclear. This study aims to investigate the therapeutic effect of PWP on the aggravation of colonic inflammation induced by a high-fat diet and particularly focuses on its regulatory mechanisms on gut microbiota, which are closely related to UC.

**Methods:**

Network pharmacology analysis was employed to screen potential pharmacological targets of PWP for UC. Histological changes in colonic tissue were observed using hematoxylin and eosin (H&E) staining, and immunofluorescence staining was performed to evaluate the expression of tight junction proteins (ZO1 and Occludin). Western blotting was used to detect the expression levels of proteins related to the PI3K/AKT/mTOR pathway, ZO1, and Occludin. qRT-PCR was conducted to measure the relative expression of inflammatory cytokines (IL-1β, IL-17, IL-6, and TNF-α) in colonic tissue. Additionally, 16S rDNA sequencing was performed to analyze gut microbiota alterations, and GC/MS was used to quantify short-chain fatty acids (SCFAs) in gut contents. The gutMgene database was utilized to validate the mediating roles of gut microbiota metabolites in the pharmacological effects of PWP. And their mediating role in PWP efficacy was verified by fecal microbiota transplantation (FMT) and butyrate supplementation.

**Results:**

Network pharmacology analysis predicted that PWP may regulate the PI3K/AKT pathway to exert therapeutic effects in UC. Experimental validation showed that PWP significantly downregulated the levels of PI3K, pAKT/AKT, and pmTOR/mTOR in colonic tissue, thereby enhancing autophagy in colonic epithelial cells, as evidenced by decreased levels of P62 and increased LC3B-II/LC3B-I ratios. Furthermore, 16S rDNA sequencing combined with targeted SCFAs analysis of gut contents revealed that the pharmacological effects of PWP may be mediated by increasing the abundance of SCFAs-producing gut microbiota (*Alistipes* and *Parabacteroides*) and elevating the levels of SCFAs in the gut.

**Conclusion:**

PWP enhances the abundance of SCFAs-producing bacteria (*Alistipes* and *Parabacteroides*) in the gut, increases the levels of butyrate, and inhibits the PI3K/AKT/mTOR pathway in the colon. These effects promote colonic autophagy and contribute to the resolution of colonic inflammation.

## Graphical Abstract

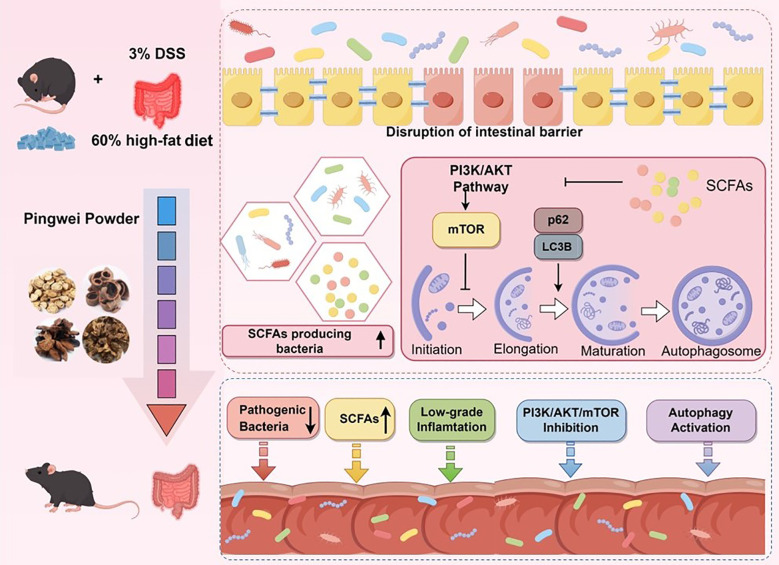

## 1 Introduction

Ulcerative colitis (UC) is an autoimmune disease characterized by symptoms including diarrhea, hematochezia, and rectal bleeding. As of 2023, the global prevalence of UC has been reported to be 5 million cases (Wangchuk et al., 2024). Research on the etiology of UC is still unclear, with genetic factors, environmental factors and dietary habits all thought to be involved in the development of UC (Wang et al., 2024a). Among other things, an increasingly large number of studies in recent years have focused on the impact of high-fat diets (Fritsch et al., 2021) and its subsequent induction of obesity (Hyun, 2021) on the pathogenesis of UC. However, current therapeutic options for UC remain constrained, primarily due to the limited efficacy of conventional medications such as 5-aminosalicylic acid (5-ASA) and Sodium-2-acetoxybenzoate (SAPA), as well as the prohibitive costs associated with biologics, including anti-tumor necrosis factor agents and anti-adhesion molecules (Gao et al., 2019). In recent years, herbal medicines have attracted considerable attention in the management of UC due to their cost-effectiveness and demonstrated efficacy, including *Scutellaria baicalensis* (Li et al., 2022), *Coptidis Rhizoma* (Xie et al., 2024), and *Reynoutria japonica* (Jiang et al., 2025), have been employed in the management of UC, demonstrating substantial therapeutic effects.

Pingwei Powder (PWP), first documented in the *Taiping Huimin Heji Jufang* (Formulas from the Imperial Pharmacy for Universal Relief), a seminal formulary of the Song Dynasty, comprises Cang Zhu (*Atractylodes lancea (Thunb.) DC.)*, Hou Po (*Magnolia officinalis Rehder & E.H. Wilson)*, Chen Pi (*Citrus reticulata Blanco)*, and Gan Cao (*Glycyrrhiza uralensis Fisch)*. It has a historical application spanning over a thousand years in the treatment of diarrhea, within TCM. Modern pharmacological studies have demonstrated that PWP, along with its constituent herbs, exhibits anti-inflammatory effects (Lee et al., 2023), facilitates the repair of intestinal mucosal barriers (Qu et al., 2022a), and regulates gut microbiota (Fan et al., 2023; Zhang et al., 2023). These properties have consequently facilitated its application in the management of UC (Zhang et al., 2019; Qu et al., 2022).

The PI3K/AKT/mTOR signaling pathway is closely associated with the pathogenesis of UC. In this study, we initially employed network pharmacology to demonstrate that PWP inhibits the activation of the PI3K/AKT/mTOR axis, a finding that was further validated through animal experiments. Additionally, we found that the inhibition of the PI3K/AKT/mTOR axis promotes the formation of autophagy in colonic epithelial cells of UC mice, thereby aiding in the control of inflammation. Given that the active ingredients of traditional herbal medicines exert their effects through the gastrointestinal tract, we also examined the impact of PWP on gut microbiota in UC mice. This study revealed that treatment with PWP not only downregulated the abundance of conditionally pathogenic bacteria, such as *Staphylococcus* and *Desulfovibrio*, in the intestines of UC mice, but also upregulated the levels of short-chain fatty acids (SCFAs)-producing bacteria and increased the concentrations of butyric acid, valeric acid, and isovaleric acid. These changes subsequently inhibited the activation of the PI3K/AKT/mTOR pathway, enhanced autophagy levels in intestinal tissues, and mitigated intestinal inflammatory injury. The role of gut microbiota and its metabolite butyrate was verified in subsequent Fecal microbiota transplantation (FMT) and butyrate supplementation experiments. Overall, our findings suggest that PWP contributes to the maintenance of intestinal mucosal integrity by upregulating SCFAs-producing bacteria and Butyrate levels, as well as modulating the PI3K/AKT/mTOR pathway to enhance autophagy in colonic epithelial cells, ultimately reducing intestinal inflammation and alleviating damage to the gut barrier.

## 2 Method

### 2.1 Reagents and materials

The herbal materials for PWP, specifically *Citrus reticulata Blanco*, *Atractylodes lancea, Magnolia officinalis*, and *Glycyrrhiza uralensis Fisch*, were procured from the First Affiliated Hospital of Jinan University in Guangzhou, China. The specific product batch numbers are as follows: *Glycyrrhiza* (HX24C01), *Magnolia* (HX24AC01), *Atractylodes* (HX25MC01), and *Citrus reticulata* (HX24C01). Dextran sulfate sodium (DSS) salt was procured from MP Biomedicals Co., Ltd. in the United States. The fecal occult blood detection kit was procured from Yuanye Bio-Technology Co., Ltd. in Shanghai, China. The high-fat diet (60% fat content) was procured from Charles River Laboratories in Shunde, China. Sodium butyrate (S817488), neomycin (N799581), ampicillin (A800429), vancomycin (V871983) and metronidazole (M813526) were purchased from Macklin, China. Mouse Interleukin 1β(IL-1β) Quantification Kit was purchased from Nanjing BYabscience Biotechnology Co.

### 2.2 Preparation of PWP decoction

The four herbal materials—*Atractylodes lancea* (24 g), *Magnolia officinalis* (18 g), *Citrus reticulata Blanco* (18 g), and *Glycyrrhiza uralensis Fisch* (12 g)—were soaked in distilled water at a ratio of 10:1 (water to herb weight) for 30 minutes, followed by boiling for an additional 30 minutes. Following the initial extraction, a second extraction was conducted using an equal volume of distilled water. The extracts from both extractions were combined and concentrated under reduced pressure at 70°C to achieve a specific final volume.

### 2.3 Identification of the medicinal components of PWP

Chromatographic analysis was conducted using an Agilent SB-C18 column (2.1 mm × 100 mm, 1.8 µm, at 40°C) with a 2 µL injection volume. Mobile phase A consisted of ultrapure water, while mobile phase B comprised acetonitrile (both with 0.1% formic acid added), and the flow rate was maintained at 0.35 mL/min. The gradient elution program was as follows: at 0.00 min, 5% B; at 9.00 min, B was linearly increased to 95% and maintained for 1 min; from 10.00 to 11.10 min, B was returned to 5% and held at that concentration until 14 min. The mass spectrometer was operated using an electrospray ionization (ESI) source at a temperature of 500°C, with the ion spray voltage (IS) set to 5500V (positive mode) and -4500 V (negative mode). The gas flow rates were maintained at 50 psi for Gas I, 60 psi for Gas II, and 25 psi for Curtain Gas. The collision-induced dissociation parameters were adjusted to a high setting. Compound identification was achieved by comparing the data against a self-constructed molecular weight database (MWDB, Metware Database).

### 2.4 Animal models and treatment

A total of thirty-five 6-week-old C57BL/6J mice (SPF grade) were procured from Charles River Laboratories in Shunde, China. The mice were randomly divided into five experimental groups. All mice were housed in an environment with a temperature of 25°C and a humidity of 60%. All groups, except for the control group, were fed a high-fat diet consisting of 60% fat content, while the control group received a standard diet. Beginning on day 15, 3% DSS was administered via the drinking water for a duration of 7 days. Furthermore, the PWP high-dose group (PWP-H), low-dose group (PWP-L), and the 5-ASA group were treated with their respective concentrations of medication (10 mL/kg): herbal dosages of 18.72 g/kg (PWP-H) and 9.36 g/kg (PWP-L), as well as 5-ASA administered via gavage at 100 mg/kg. Both the control and model groups received saline via gavage. The Animal Experiment Protocol listed below has been reviewed and approved by Laboratory Animal Welfare and Ethics Committee of Jinan University under approved protocol number IACUC-20241115-08.

### 2.5 Fecal microbiota transplantation and butyrate intervention

The construction of pseudo-germ-free mice was based on a previously established methodology (Li et al., 2021). Briefly, a cocktail of antibiotics was administered via drinking water for 14 consecutive days. Subsequently, 3% dextran sodium sulfate (DSS) was added to the drinking water of all mice to induce colonic inflammation. In addition, sodium butyrate was added to the drinking water of the ABX-PWP-BA group to achieve a final concentration of 200 mM. FMT interventions were performed as follows: the FMT-Mol group received fecal enemas from the model group, while the FMT-PWP group received fecal enemas from the PWP-H treatment group. Furthermore, the ABX-PWP and ABX-PWP-BA groups were subjected to intragastric administration of PWP herbal medicine at a dose of 18.72 g/kg once daily for 7 days.

### 2.6 Body weight and disease activity index

Body weight measurements were conducted on days 17, 19, and 21. Fecal consistency was assessed, and fecal samples were collected for fecal occult blood analysis. The Disease Activity Index (DAI) scores were calculated based on established methodologies as described in the literature (Li et al., 2022).

### 2.7 Histopathological observation

Colon tissue were fixed in 4% paraformaldehyde for 24 hours. Fixed tissues were dehydrated in graded ethanol (70%, 80%, 90%, 100%), followed by xylene for transparency. Tissues were embedded in paraffin wax and sectioned at 4µm thickness using a microtome. Sections were mounted on glass slides and dried. For H&E staining, sections were deparaffinized in xylene, rehydrated through graded ethanol (100%, 95%, 70%), and rinsed in distilled water. Nuclei were stained with hematoxylin for 3 minutes, followed by differentiation in citrate buffer (pH 4.8) for 10 seconds. Cytoplasm was counterstained with eosin for 1 minutes. Sections were dehydrated, cleared in xylene, and mounted with neutral mounting medium. Slides were examined under a light microscope for evaluation of tissue morphology.

### 2.8 Immunofluorescence assay

After antigen retrieval of colon paraffin sections, the sections were placed in a mixture of primary antibodies ZO1 (GB111401, Servicebio, China) and Occludin (GB151981, Servicebio, China), and incubated at 4°C for 12 hours. Subsequently, secondary antibodies (GB23303, Servicebio, China) (GB21303, Servicebio, China) were incubated at room temperature in the dark for 1 hour, followed by DAPI staining of the cell nuclei before imaging.

### 2.9 Elisa assay for serum IL-1β levels

Serum IL-1βconcentrations were measured using a commercial mouse IL-1βELISA kit (BYabscience, BY-EM220174) according to the manufacturer’s protocol. Briefly, standards and samples were added to antibody-coated 96-well plates and incubated with detection reagent for the recommended times, followed by wash steps to remove unbound material. After addition of substrate solution and termination of the enzymatic reaction, optical density was read at 450 nm using a microplate reader. Concentrations were calculated using a standard curve generated with SigmaPlot 15.0 software.

### 2.10 Western blotting

Colonic tissues were collected and lysed using high-strength RIPA lysis buffer (supplemented with protease and phosphatase inhibitors) to extract proteins. Following protein quantification, the samples were combined with the loading buffer. Proteins were separated using 10% SDS-PAGE and subsequently transferred to a PVDF membrane. The membrane was incubated with the primary antibody overnight, followed by incubation with the secondary antibody. Protein bands were visualized by chemiluminescence. Image J software was utilized to analyze the density of the protein bands.

### 2.11 Quantitative real-time PCR

Colonic tissues were collected, and RNA was extracted using the SteadyPure Universal RNA Extraction Kit (Accurate Biotechnology Co., Ltd; Hunan, China), followed by reverse transcription into cDNA using the Evo M-MLV RT Mix Kit (Accurate Biotechnology Co., Ltd; Hunan, China). Primers for the target genes were designed (see supplementary table), and qRT-PCR was performed. Relative quantification was determined using the 2^−ΔΔCT^ method with Gapdh as the internal reference gene.

### 2.12 Fecal 16S rDNA sequencing

Mice were euthanized, and cecal contents were promptly collected in 1.5 mL centrifuge tubes, which were subsequently stored in liquid nitrogen before being transferred to -80°C for long-term storage. Total DNA was extracted from fecal samples, and the concentration and purity of the DNA were assessed. The V3 and V4 regions of the 16S rDNA gene were amplified using specific primers. Following purification, quantification, and normalization of the amplification products, sequencing libraries were constructed and sequenced using the Illumina NovaSeq PE250 platform for 16S rDNA amplicon sequencing. Microbiota sequencing and data analysis were performed by APExBIO (Houston, USA).

### 2.13 GC/MS detection of intestinal SCFAs

Standard solutions of acetic acid, propionic acid, butyric acid, isobutyric acid, valeric acid, isovaleric acid, and caproic acid were prepared at a concentration of 100 mg/mL. The internal standard, 4-methylpentanoic acid, was prepared in ether at a concentration of 75 µg/mL. Working standard solutions of varying concentrations (0.02 µg/mL to 100 µg/mL) were prepared for gas chromatography-mass spectrometry (GC/MS) calibration. Fecal samples, measuring 200 µL, were mixed with 50 µL of 15% phosphoric acid, 100 µL of the internal standard solution, and 400 µL of ether. The mixture was homogenized for 1 minute, subsequently centrifuged at 12,000 rpm for 10 minutes at 4°C, and the supernatant was analyzed.

GC/MS analysis was conducted using a Thermo Trace 1300 system (Thermo Fisher Scientific, USA) equipped with an Agilent HP-INNOWAX capillary column (30 m × 0.25 mm ID × 0.25 µm). A 1 µL sample was injected at a 10:1 split ratio. The initial temperature was set at 90°C and ramped to 120°C at 10°C/min, then to 150°C at 5°C/min, and finally to 250°C at 25°C/min, where it was held for 2 minutes. The carrier gas used was helium, with a flow rate of 1 mL/min. Mass spectrometry was conducted utilizing a Thermo ISO 7000 mass spectrometer (Thermo Fisher Scientific, USA) operating in electron impact (EI) mode with an electron energy of 70 eV and in selected ion monitoring (SIM) scanning mode.

### 2.14 Transmission electron microscopy

Colonic tissues were promptly fixed using an electron microscopy fixative at 4°C for 24 hours in the dark. The tissues were subsequently exposed to 1% osmium tetroxide for 2 hours, followed by a gradient dehydration process in ethanol at room temperature. The samples were embedded in 812 resin and polymerized at 60°C for a duration of 48 hours. Ultra-thin sections, measuring 60–80 nm, were stained with 2% uranyl acetate in alcohol for 8 minutes, followed by three washes with 70% ethanol and three washes with ultra-pure water. Finally, the sections were stained with 2.6% lead citrate for 8 minutes in a CO_2_-free environment and subsequently washed again with ultra-pure water. The sections were dried overnight at room temperature and subsequently observed using a Transmission Electron Microscope (TEM).

### 2.15 Potential therapeutic targets of PWP in the treatment of UC

#### 2.15.1 Collection of active ingredients and targets of PWP

Using the TCM Systems Pharmacology Database and Analysis Platform (TCMSP, https://old.tcmsp-e.com/tcmsp.php), we identified the bioactive compounds present in the four herbal ingredients of PWP: Atractylodes lancea., Magnolia officinalis, Citrus reticulata Blanco, and Glycyrrhiza uralensis Fisch. The selection criteria included an oral bioavailability (OB) threshold of ≥ 30% and drug-likeness (DL) threshold of ≥ 0.18. The protein targets corresponding to these bioactive compounds were retrieved and standardized to gene names through the UniProt database (https://www.uniprot.org).

#### 2.15.2 Collection of UC-associated targets

The UC-associated targets were identified using “UC” as a keyword in the OMIM database (https://www.omim.org/), DrugBank (https://go.drugbank.com/), TTD (http://db.idrblab.net/ttd), and GeneCards (https://www.genecards.org/). The targets obtained from these databases were consolidated, and duplicates were eliminated to create a comprehensive catalog of UC-associated targets.

#### 2.15.3 Construction of protein-protein interaction network

To identify interactions between the therapeutic targets of PWP and those associated with UC-related diseases, the common targets were calculated using the Venn Diagram platform (http://www.interactivenn.net). The identified common targets were subsequently uploaded to the STRING database (https://cn.string-db.org/) to construct a PPI network for further analysis.

#### 2.15.4 KEGG pathway enrichment analysis

To investigate the potential mechanisms through which PWP may treat UC, the common targets were submitted to the Metascape platform (https://metascape.org) for KEGG pathway enrichment analysis. The analysis was conducted using Homo sapiens as the chosen species. The enrichment results were visualized using a bubble chart generated by the Bioinformatics platform (http://www.bioinformatics.com.cn).

#### 2.15.5 Construction of the PWP therapeutic target and UC disease target-pathway network

A comprehensive network integrating the therapeutic targets of PWP, UC disease targets, and enriched pathways was constructed using Cytoscape version 3.7.2. Network topology parameters, including degree, betweenness centrality, and closeness centrality, were analyzed utilizing the built-in tools of Cytoscape. The key active components and core targets contributing to the therapeutic effects were identified based on the analysis of these topological parameters.

### 2.16 Interaction analysis of SCFAs with UC-associated PI3K/AKT/mTOR and autophagy-related genes based on gutMgene database

Target Identification of gut microbiota metabolites: metabolites and their corresponding murine gut targets were retrieved from the gutMGene database (http://bio-annotation.cn/gutmgene/). The SCFAs detected in intestinal samples via GC/MS (acetic acid, propionic acid, isobutyric acid, butyrate, isovaleric acid, valeric acid, and caproic acid) were matched to their corresponding murine metabolite entries in the gutMGene database to identify associated molecular targets.

Intersection with key signaling pathways: The identified targets of the seven SCFAs were compared with genes annotated in the UC-associated PI3K/Akt/mTOR and autophagy signaling pathway and the autophagy pathway (KEGG pathway annotations).

Network visualization: The overlapping targets, their associated SCFAs, and the corresponding pathways were imported into Cytoscape version 3.9.1 to construct a metabolite-target-pathway interaction network.

### 2.17 Statistical analysis

Statistical analyses were conducted using SPSS version 25.0, and the results were expressed as the mean ± standard deviation (SD). Differences among groups were assessed using a one-way analysis of variance (ANOVA). *Post-hoc* multiple comparisons were performed using the LSD method for homoscedastic data or the Tamhane’s T2 method for heteroscedastic data. Statistical significance was defined as follows: Control group versus Model group: #: P<0.05, ##: P<0.01; Model group versus PWP group: *: P< 0.05, **: P<0.01.

Correlation analysis of gut microbiota abundance with SCFAs metabolites, colon tissue cytokines expression and autophagosome number was carried out using the spearman correlation calculation method. This process was performed using the Metware Cloud, a free online platform for data analysis (https://cloud.metware.cn).

## 3 Result

### 3.1 Qualitative analysis of the components of PWP

**Figure 1:**
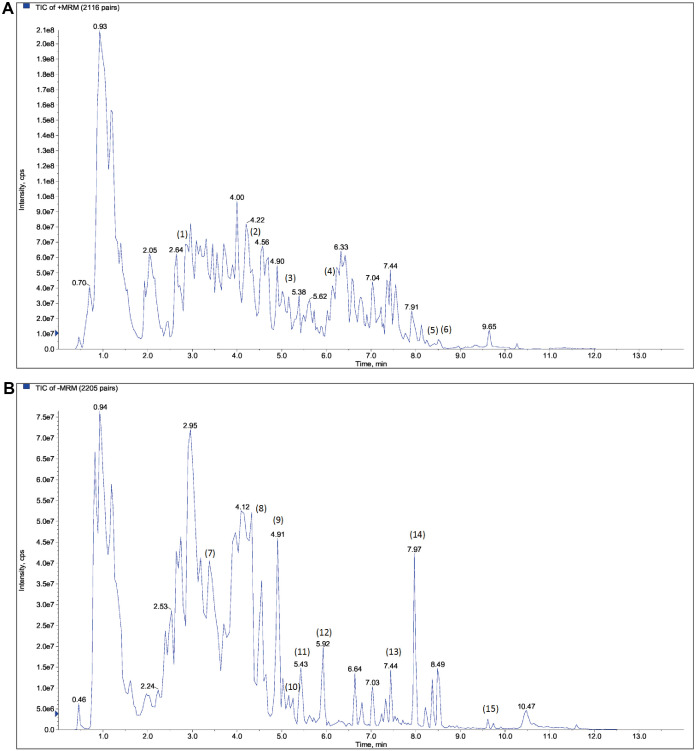
Qualitative analysis of the components of PWP based on UPLC/MS/MS detection combined with Metware database comparison. **(A)** cation mode; **(B)** anion mode.

The PWP was analyzed using UPLC/MS/MS, resulting in the detection of 3,940 compounds (**Supplementary Table 1**). Based on secondary spectral information matched with the MWDB, we identified and characterized 15 quality control or key constituents derived from the four herbal components of PWP (*Atractylodis Rhizoma*, *Magnoliae Officinalis Cortex*, *Citri Reticulatae Pericarpium*, and *Glycyrrhizae Radix*) (**Table 1**, **Figure 1**).

**Table 1:** Characteristic compounds of PWP.

	Compounds	Formula	Retention time (min)	Ionization model
1	Atractyloside A	C21H36O10	2.8	[M+H]+
2	Neohesperidin	C28H34O15	4.4	[M+H]+
3	Liquiritigenin	C15H12O4	5.1	[M+H]+
4	Isoliquiritigenin	C15H12O4	6.1	[M+H]+
5	Atractylenolide II	C15H20O2	8.5	[M+H]+
6	3β-Hydroxyatractylon	C15H20O2	8.6	[M+H]+
7	Magnoloside A	C29H36O15	3.3	[M-H]-
8	Hesperidin	C28H34O15	4.3	[M-H]-
9	Atractyloside G	C21H36O8	4.9	[M-H]-
10	Magnatriol B	C15H14O3	5.2	[M-H]-
11	Magnolignan A	C18H20O4	5.4	[M-H]-
12	Magnolignan E	C18H18O4	5.8	[M-H]-
13	Atractylenolide III	C15H20O3	7.4	[M-H]-
14	Honokiol	C18H18O2	7.9	[M-H]-
15	Hypoglycyrrhizic acid (β)	C30H46O4	9.7	[M-H]-

### 3.2 The therapeutic effect of PWP on UC in mice

**Figure 2:**
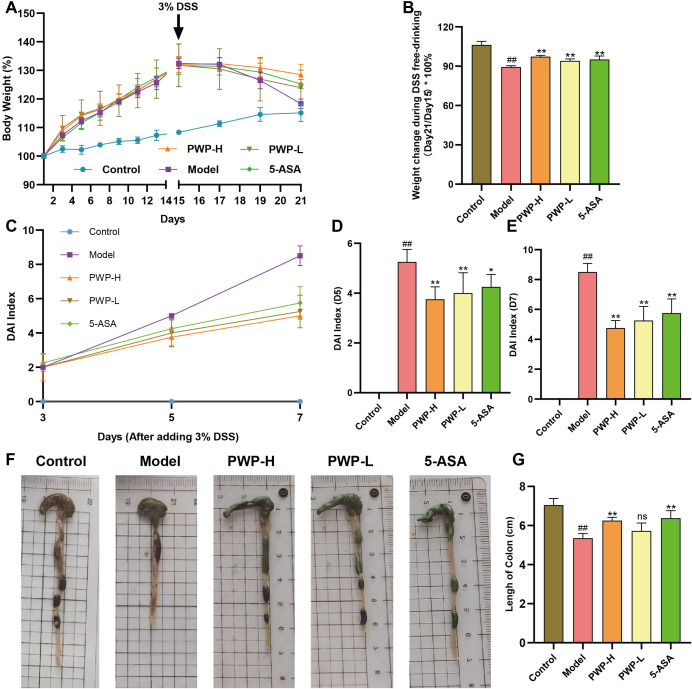
Improvement of general condition of UC mice by PWP. **(A)** Line graph of weight change; **(B)** Weight at the end of the experiment (Day 21); **(C–E)** Changes in the DAI; **(F)** Appearance of the colon; **(G)** The length of colon. Control group versus Model group: ##: *P*<0.01; Model group versus PWP group: *P< 0.05, **P<0.01, ns, not significant.

We developed a mouse model using 3% DSS combined with a high-fat diet, and assessed the successful establishment of the model by utilizing 5-ASA as a positive control drug. Regarding body weight, following the introduction of 3% DSS in drinking water from day 15, overall body weights exhibited a decreasing trend. To assess body weight changes during the period of being given DSS free drinking (Day15-Day21), we compared the percentage of body weight change in each group of mice compared to that prior to the administration of DSS, and the results showed a significant reduction in the model group of mice compared to the control group (*P*<0.01). Following treatment with either 5-ASA or PWP, the degree of weight loss was less than that observed in the model group, particularly in the PWP-H group, which demonstrated a significant improvement compared to the model group (*P*<0.05) (**Figures 2A, B**). The DAI effectively reflected disease progression in the UC model, and treatment with 5-ASA significantly reduced the DAI on the fifth day of DSS administration (*P*<0.05), with a more pronounced reduction observed after treatment with PWP (*P*<0.01). On the seventh day, the DAI was significantly reduced (*P*<0.01) in all treatment groups compared to the model group (**Figures 2C–E**). Colon length serves as a reflective indicator of intestinal inflammation, with shorter lengths predicting more severe colonic inflammation. Compared to the control group, all groups subjected to UC modeling exhibited reduced colon lengths, with the model group showing the most significant reduction (*P*<0.01) and exhibiting bloody stool-like changes in intestinal contents. Conversely, both the 5-ASA and PWP-H treatment groups significantly increased colon length (*P*<0.01) (**Figures 2F, G**).

### 3.3 PWP repairs intestinal barrier damage and reduces inflammation of the colon tissue

**Figure 3:**
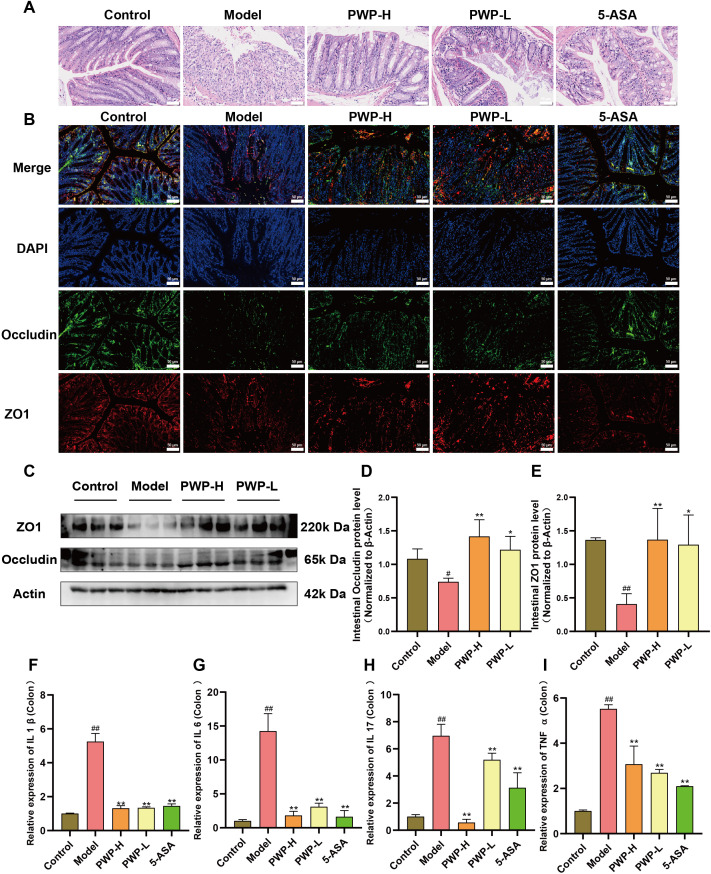
PWP attenuates colonic tissue destruction and reduces inflammation. **(A)** H&E staining of colon tissue; **(B)** Immunofluorescent staining for colonic tissue tight junction proteins (ZO1, Occludin); **(C–E)** Western blot analysis of colonic tissue tight junction proteins; **(F–I)** Levels of mRNA expression of pro-inflammatory cytokines in colonic tissues. Control group versus Model group: #: *P*<0.05, ##: *P*<0.01; Model group versus PWP group: *P< 0.05, **P<0.01.

Disruption of the intestinal barrier is a characteristic pathological manifestation of UC. Histological examination of HE-stained colon sections revealed significant disruption of the villous structure of colonic epithelial cells in the model group, accompanied by extensive infiltration of neutrophils and macrophages compared to the control group. In contrast, the villous structure of the colonic epithelium remained intact, and inflammatory cell infiltration was attenuated in the groups treated with PWP or 5-ASA (**Figure 3A**). Immunofluorescence analysis demonstrated that the fluorescence intensity of ZO1 and Occludin, tight junction proteins found in colonic epithelial cells, was attenuated in the model group. Conversely, treatment with 5-ASA and PWP at both high and low doses significantly up-regulated the fluorescence intensity of these intestinal tight junction proteins (**Figure 3B**). Further examination of colonic tissues via Western blot analysis revealed that ZO1 and Occludin were significantly down-regulated in the colonic tissues of model mice, whereas their expression levels were restored following PWP treatment (*P*<0.05) (**Figures 3C–E**). In the model group, damage to the intestinal barrier was associated with a tissue inflammatory response, as evidenced by the up-regulation of pro-inflammatory factors IL-1β, IL-17, IL-6, and TNF-α (*P*<0.01). In contrast, treatment with PWP significantly down-regulated the expression of these pro-inflammatory factors in colonic tissues, particularly in the high-dose group (*P*<0.01) (**Figures 3F–I**).

### 3.4 Target prediction and PPI analysis of PWP in UC treatment

Using the criteria of OB ≥ 30% and DL ≥ 0.18, a total of 104 active compounds of PWP were identified from the TCMSP database. Specifically, 43 targets were identified for *Atractylodes lancea*, 65 for *Citrus reticulata Blanco*, 25 for *Magnolia officinalis*, and 225 for *Glycyrrhiza uralensis Fisch*. After removing duplicates, a total of 235 unique pharmacological targets for PWP were obtained.

**Figure 4:**
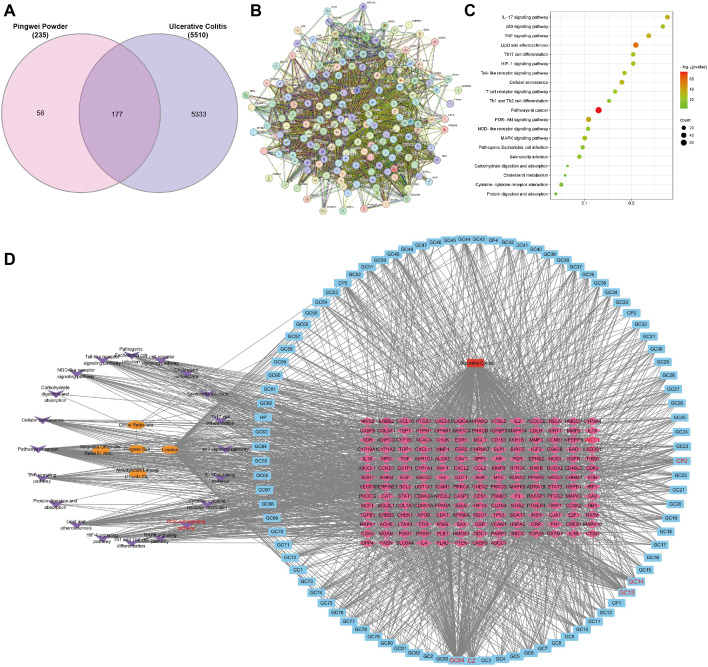
Target prediction and PPI analysis of PWP in UC treatment. **(A)** Venn diagram of the intersection of PWP drug targets and disease targets in UC; **(B)** PPI network diagram of overlapping targets; **(C)** KEGG pathway enrichment analysis of overlapping targets; **(D)** Network topology analysis of PWP in the treatment of UC.

A comprehensive search for UC-related targets across the OMIM, DrugBank, TTD, and GeneCards databases was conducted, identifying 5,510 disease-associated targets. The intersection of PWP targets and UC disease targets yielded 177 potential therapeutic targets for PWP, which were visualized using a Venn diagram (**Figure 4A**). PPI networks for these 177 targets were constructed using the STRING platform (**Figure 4B**).

KEGG pathway enrichment analysis of the intersected targets revealed significant involvement in pathways including the IL-17 signaling pathway, p53 signaling pathway, Toll-like receptor signaling pathway, and PI3K/AKT signaling pathway (**Figure 4C**). Further network topology analysis using Cytoscape version 3.7.2 identified the core components and key targets involved in the therapeutic mechanisms of PWP. The constructed network consisted of 294 nodes and 1,990 edges, with dense connections highlighting the multi-component, multi-target, and multi-pathway therapeutic effects of PWP in the treatment of UC. This analysis demonstrated that the active components of PWP exert therapeutic effects on UC by interacting with distinct targets through various pathways (**Figure 4D**).

### 3.5 PWP inhibits activation of PI3K/AKT/mTOR pathway related proteins in colon tissues

**Figure 5:**
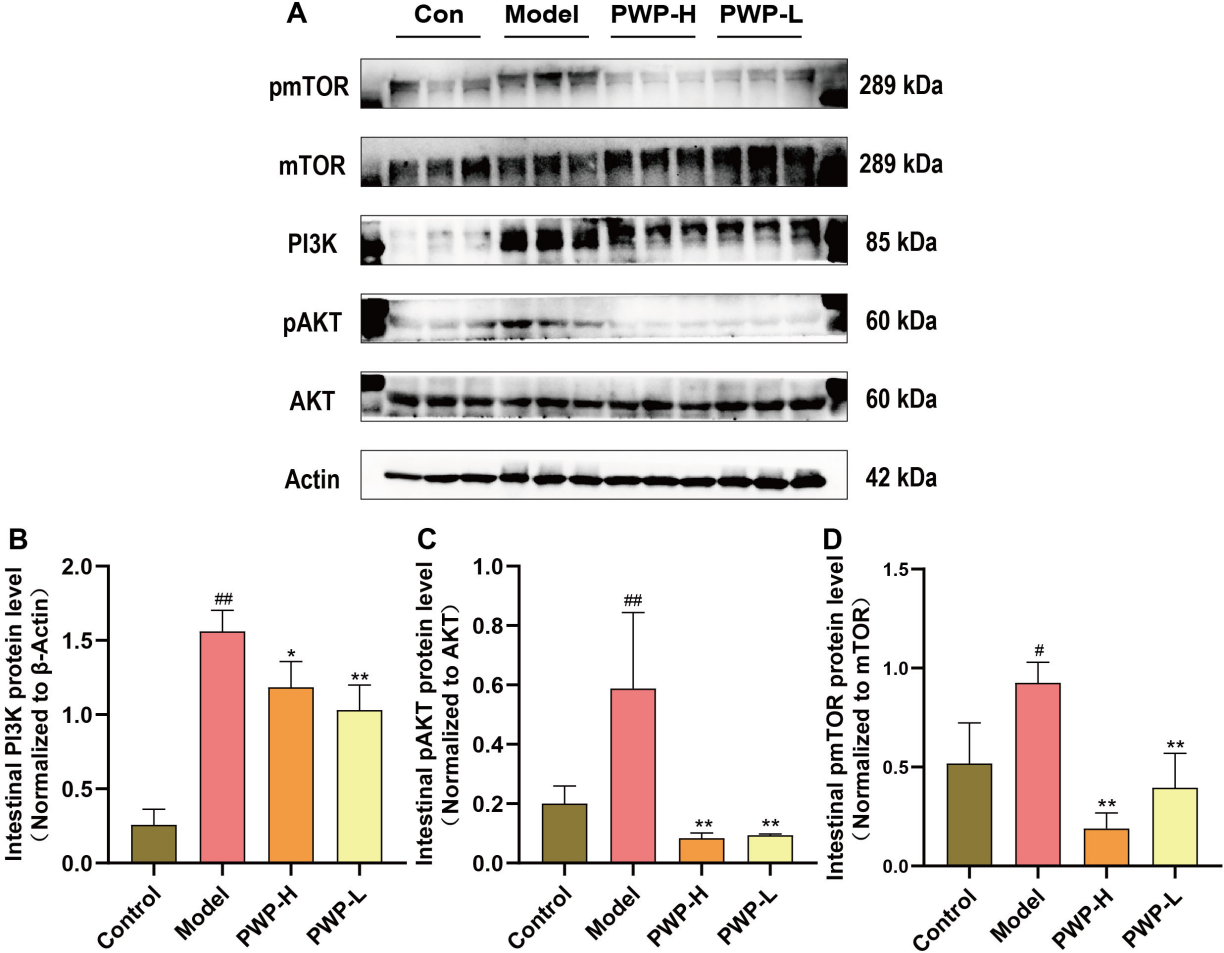
PWP inhibits activation of the PI3K/AKT/mTOR signaling pathway in the colonic tissues. **(A)** Western blot bands; **(B)** Colonic PI3K protein levels; **(C)** pAKT/AKT ratio in colonic tissue; **(D)** pmTOR/mTOR ratio in colonic tissue. Control group versus Model group: #: *P*<0.05, ##: *P*<0.01; Model group versus PWP group: *P< 0.05, **P<0.01.

Injury to the intestinal mucosa and the entry of antigenic substances from the intestinal lumen into the lamina propria can trigger the activation of the PI3K/AKT/mTOR pathway. The preceding network pharmacological analyses suggest that PWP may treat UC by modulating the PI3K/AKT-related pathway. The pattern of phosphorylation serves as an indicator of the activation state of these proteins. PI3K was significantly upregulated in the model group compared to normal controls (*P*<0.01); correspondingly, the pAKT/AKT and pmTOR/mTOR ratios were also higher in the model group (*P*<0.01). Following treatment with PWP, both the PI3K (*P*<0.01) and pAKT/AKT (*P*<0.05) ratios were significantly down-regulated. Meanwhile, a significant down-regulation of the pmTOR/mTOR ratio was observed in the PWP-H group (*P*<0.01) (**Figures 5A–D**). These findings suggest that the PI3K/AKT/mTOR pathway is significantly activated in the disease state, and that PWP inhibits the activation of proteins within this pathway, which may be a key mechanism for its efficacy.

### 3.6 PWP promotes autophagy levels in colonic tissues

**Figure 6:**
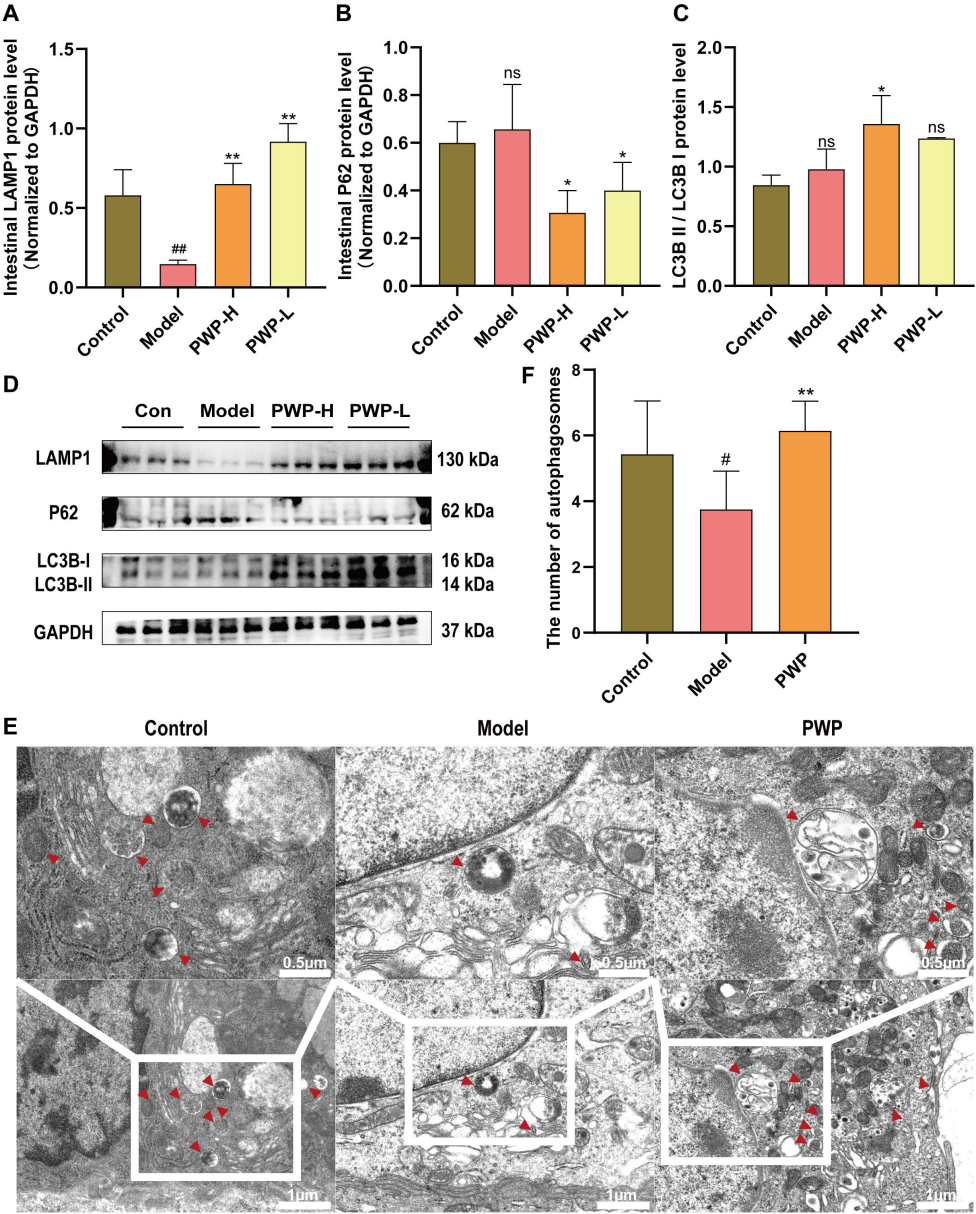
PWP alleviates intestinal inflammation by upregulating autophagy. **(A–D)** Western blot of LAMP1, P62 and LC3B-II/LC3B-I and its relative expression analysis; **(E)** Colonic tissue ultrastructure visualized by TEM.; **(F)** Number of autophagosomes observed via TEM. Control group versus Model group: #: *P*<0.05, ##: *P*<0.01; Model group versus PWP group: *P< 0.05, **P<0.01, ns, not significant.

mTOR is an inhibitory target for initiating the autophagy process. The up-regulated pmTOR/mTOR ratio in the colonic tissues of the model group is detrimental to the macro-autophagy process of these tissues, impeding the phagolysis and catabolism of damaged molecules. During the autophagy process, lysosomal-associated membrane protein 1 (LAMP1) serves as a structural component of lysosomal membranes, facilitating the fusion of autophagosomes with lysosomes during the autophagic process, and its expression level was significantly upregulated after treatment of PWP (*P*<0.01) (**Figure 6A**). P62 is consumed as a substrate, and the expression level of P62 in the colonic tissues of mice receiving high-dose PWP treatment was significantly down-regulated (*P*<0.05) compared to the model group (**Figure 6B**). The ratio of LC3B-II/LC3B-I reflects the level of autophagy, with higher levels of LC3B-II indicating increased autophagosome formation. Similarly, the ratio of LC3B-II/LC3B-I was down-regulated in the model group (*P*<0.05), whereas it was restored after treatment with PWP (*P*<0.05) (**Figures 6C, D**). Furthermore, TEM revealed that the control group exhibited normal morphological structures of the colon, including intact mitochondrial structures and clear endoplasmic reticulum structures without swelling or dilatation. In contrast, the model group displayed a significant reduction in the number of autophagosomes and autophagolysosomes (*P*<0.05), accompanied by mitochondrial swelling and endoplasmic reticulum dilatation. Compared to the model group, the PWP-treated group exhibited a significant increase in the number of autophagosomes and autophagolysosomes (*P*<0.01), with an increase in structurally intact mitochondria, reduced swelling, and decreased endoplasmic reticulum dilatation (**Figures 6E, F**).

### 3.7 PWP modulates the gut microbiota composition

**Figure 7:**
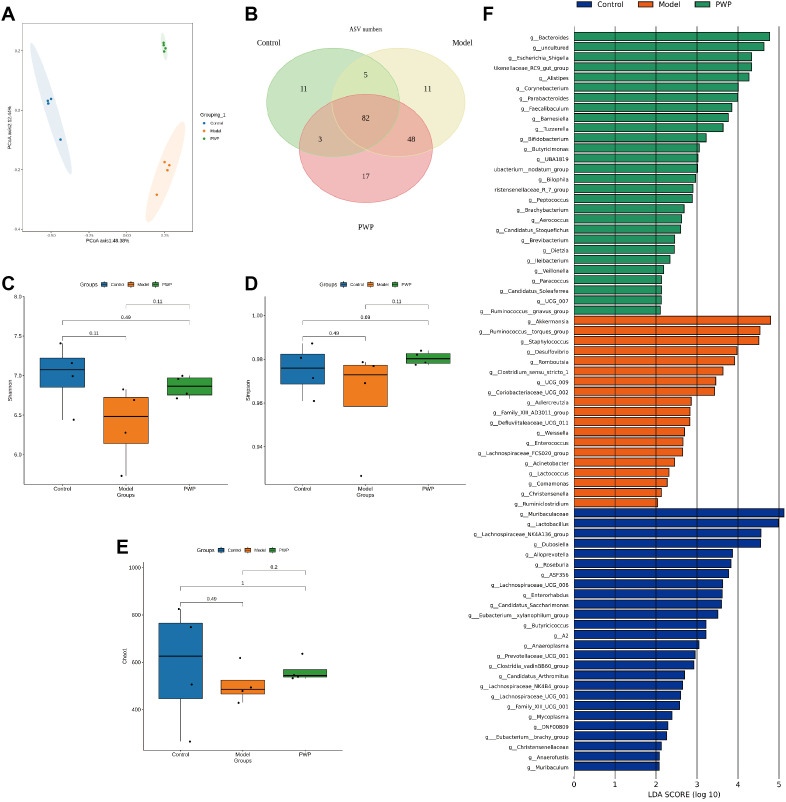
Modulation of gut microbiota by PWP. **(A)** Plot of principal coordinate analysis; **(B)** Venn plot based on the genus level of gut microbiota; **(C–E)** Plot of alpha diversity indices; **(F)** Linear discriminant analysis effect size (LEfSe) analysis. (n=4).

The gut microbiota, comprising up to 100 trillion cells, plays a critical role in various diseases, including gastrointestinal, neurological, and respiratory disorders (Wang et al., 2023, 2024b; Wu et al., 2024). Oral administration is the predominant route for herbal medicines, and modulating gut microbiota along with their metabolites serves as a key mechanism underlying their therapeutic effects. Principal coordinate analysis (PCoA) revealed significant alterations in the gut microbiota composition of UC mice, which were notably influenced by PWP treatment (**Figure 7A**). At the genus level, 82 overlapping genera were identified across the three groups, with 17 unique genera present in the UC model group and 11 unique genera emerging following PWP treatment (**Figure 7B**). Alpha diversity indices, including the Shannon and Simpson indices, reflect microbial diversity, while the Chao indices indicate species richness. Both diversity and richness indices were significantly reduced in UC model mice, but they were partially restored following treatment with PWP. However, the alpha diversity indices did not achieve statistical significance (*P* > 0.05) (**Figures 7C–E**). Linear discriminant effect size (LEfSe) analysis revealed the enrichment of opportunistic pathogens including *Staphylococcus*, *Ruminococcus* torques group, *Desulfovibrio*, *Clostridium sensu stricto 1*, and *Enterococcus* in the model group. Conversely, beneficial bacteria, including *Bifidobacterium* and *Bacteroides*, were enriched in the PWP treatment group (**Figure 7F**).

### 3.8 PWP repairs intestinal damage by regulating intestinal metabolite levels of SCFAs

**Figure 8:**
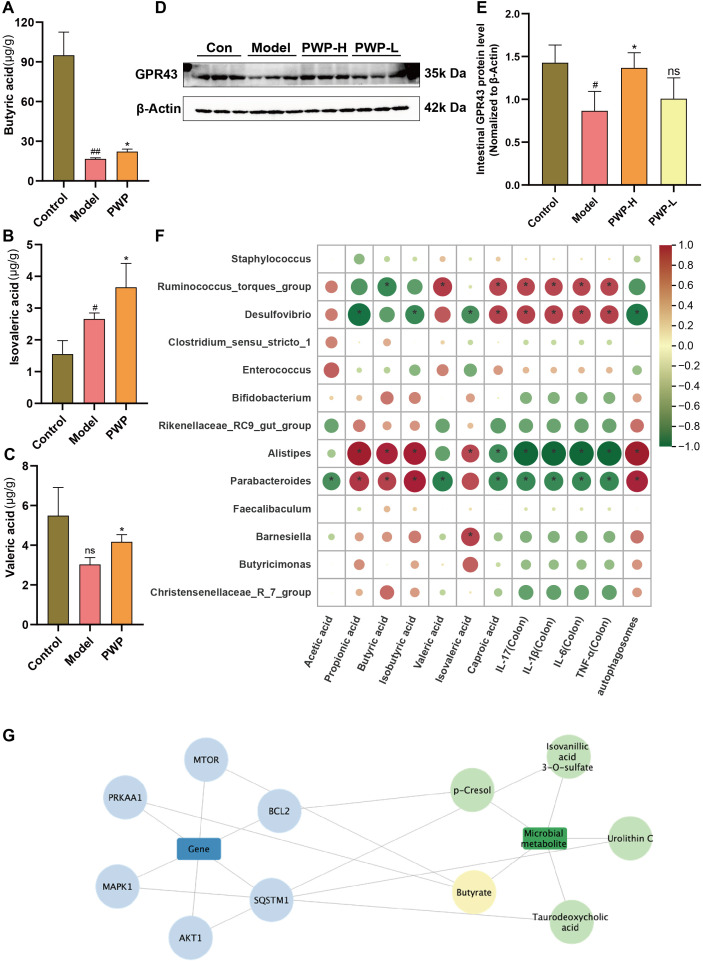
PWP inhibits the PI3K/AKT/mTOR pathway and promotes colon epithelial autophagy levels by regulating gut microbiota-SCFAs metabolites. **(A–C)** Levels of SCFAs in the gut; **(D, E)** Western blot of GPR43 and its relative expression analysis; **(F)** Spearman analysis on differential gut microbiota with SCFAs, the expression of inflammatory factors, and the number of autophagosomes; **(G)** SCFAs-associated targets in complex with UC-associated PI3K/AKT/mTOR and autophagy pathway targets. Control group versus Model group: #: *P*<0.05, ##: *P*<0.01; Model group versus PWP group: *P< 0.05, (n=4).

Based on our findings that PWP modulates known SCFAs-producing bacteria, we further quantified the levels of SCFAs in the intestinal contents. PWP treatment significantly upregulated the levels of butyric, isovaleric, and valeric acids in the gut contents compared to the model group (**Figures 8A–C**). GPR43, a recognition receptor for SCFAs, was significantly up-regulated in the PWP-treated group (**Figures 8D, E**). Further correlation analysis revealed that *Desulfovibrio*, which was enriched in the model group, was negatively correlated with fecal levels of propionic acid, isobutyric acid, and isovaleric acid, while *Ruminococcus* torques group was negatively correlated with butyric acid levels. Both were positively correlated with the levels of inflammatory factors in colonic tissue. In contrast, *Alistipes* and *Parabacteroides*, which were enriched in the feces of the PWP-treated group, were positively correlated with fecal levels of propionic acid, butyric acid, isobutyric acid, and isovaleric acid, while negatively correlated with the colonic tissue levels of IL-17, IL-6, TNF-α, and IL-1β (**Figure 8F**). A search of the gutMgene database revealed that the targets of action of SCFAs generated 10 crossover repeats in UC-associated PI3K/AKT/mTOR as well as autophagy pathway-associated targets, among which butyric acid was identified as a core target (**Figure 8G**). Correlation analysis also revealed that the abundance of *Alistipes* and *Parabacteroides* in feces was positively correlated with the number of autophagosomes formed in colonic epithelial cells, whereas *Desulfovibrio* exhibited a negative correlation. The results suggest that the upregulation of colonic autophagy levels by PWP, through the inhibition of the PI3K/AKT/mTOR pathway, may be mediated by its regulatory effects on intestinal SCFAs-producing bacteria and their associated SCFAs.

### 3.9 PWP elevates intestinal butyrate levels to suppress PI3K/AKT/mTOR axis activation and promote autophagy

**Figure 9:**
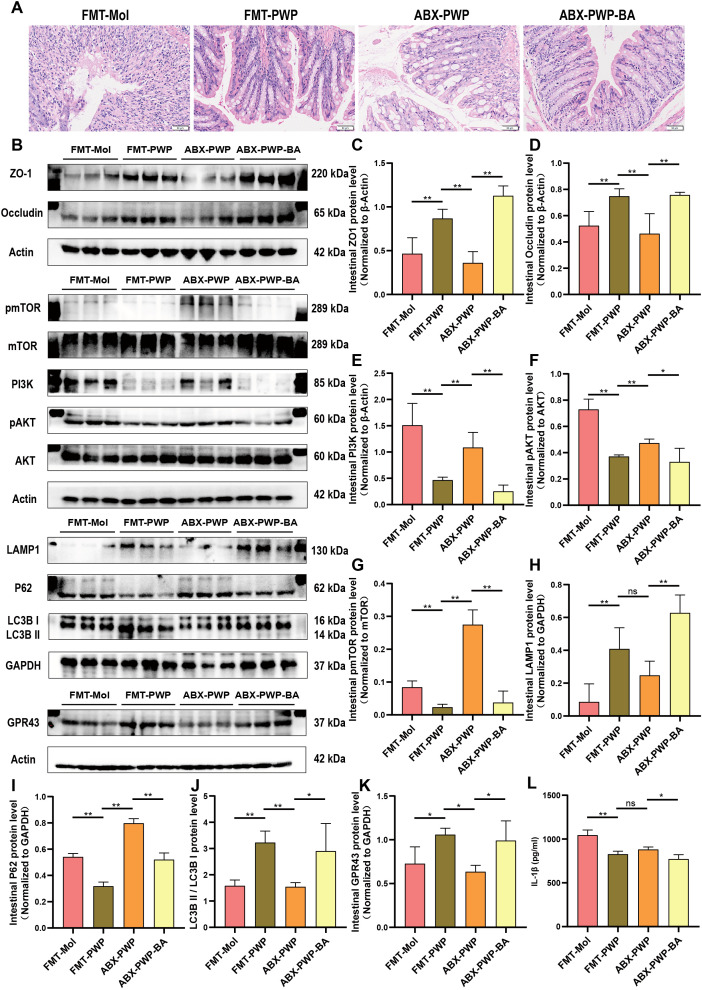
PWP modulates the gut microbiota and butyrate levels to inhibit PI3K/AKT/mTOR pathway activation and promote autophagy. **(A)** H&E staining of colon tissue; **(B)** Western blot bands; **(C–K)** Quantification of ZO-1, Occludin, PI3K, pAKT/AKT, pmTOR/mTOR, LAMP1, P62, GPR43, and LC3B II/I; **(L)** ELISA analysis of serum IL-1β levels. (*P< 0.05, **P<0.01). ns, not significant.

To further confirm that the gut microbiota serves as a central mediator of PWP’s therapeutic effects, we performed a FMT experiment. In a DSS-induced colitis model, mice receiving fecal gavage from the PWP-H treatment group—rather than those mice treated with PWP after gut microbiota clearance with antibiotics—exhibited markedly attenuated intestinal inflammatory injury (**Figure 9A**) and significantly higher expression of intestinal tight junction proteins (*P* < 0.01) (**Figures 9B–D**) compared with mice that received feces from the model group. Concurrently, activation of PI3K/AKT/mTOR pathway related proteins was significantly inhibited (*P* < 0.01) (**Figures 9E–G**), while LAMP1 and LC3BII/LC3BI levels were elevated, and the autophagy substrate P62 was more extensively consumed (*P* < 0.01) (**Figures 9H–J**).

Butyrate, a key gut microbiota derived metabolite, may represent the principal effector molecule in this process. In a butyrate supplementation experiment, mice in the ABX-PWP-BA group displayed reduced colonic inflammatory injury compared with the ABX-PWP group. This improvement was accompanied by upregulation of the SCFAs receptor GPR43 (*P* < 0.05) (**Figure 9K**) and increased expression of tight junction proteins ZO-1 and Occludin (*P* < 0.01) (**Figures 9B–D**). Additionally, butyrate supplementation suppressed phosphorylation of PI3K/AKT/mTOR pathway–related proteins (*P* < 0.01) (**Figures 9E–G**), markedly increased the expression of autophagy-related proteins LAMP1 and LC3BII/LC3BI, and further enhanced consumption of P62 (*P* < 0.05) (**Figures 9H–J**). ELISA analysis revealed that serum IL-1β levels were significantly reduced in both the ABX-PWP-BA and FMT-PWP groups (*P* < 0.05) (**Figure 9L**). Collectively, these findings indicate that during PWP treatment of HFD-induced UC, the gut microbiota and its metabolite butyrate are key intermediaries mediating its therapeutic efficacy. This mechanism contributes to suppression of PI3K/AKT/mTOR pathway activation, thereby enhancing autophagy in colonic tissue, promoting repair of the intestinal barrier, and mitigating inflammatory injury.

## 4 Discussion

Recent studies have shown that a high-fat diet exacerbates disease severity in UC, as evidenced by increased intestinal barrier damage and increased systemic inflammation (Zheng et al., 2022b; Jiang et al., 2025). A large cohort study involving 170,805 participants demonstrated that high intake of trans-unsaturated fatty acids increases the risk of Crohn’s disease and UC (Ananthakrishnan et al., 2014). Conversely, adherence to a healthy dietary pattern, such as the Mediterranean diet, helps maintain sustained clinical remission in patients with IBD (Haskey et al., 2023; Xia et al., 2024). Mechanistically, HFD has been shown to increase the abundance of intestinal non-CD1d-restricted natural killer (NK) T cells while reducing regulatory T (Treg) cell levels in UC (Ma et al., 2007). Gulhane et al. reported that HFD induces endoplasmic reticulum stress in intestinal secretory goblet cells, thereby impairing the mucus barrier and promoting colonic injury (Gulhane et al., 2016). Alterations in gut microbiota, particularly reductions in SCFAs-producing bacteria, represent a key mechanism by which HFD aggravates intestinal barrier injury in IBD (Thomas et al., 2023). SCFAs can bind to GPR43, thereby maintaining intestinal barrier integrity and alleviating inflammation (Kreuter et al., 2019).

TCM treatments for UC typically employ dampness-dispelling herbs such as *Coptis chinensis (Huanglian)*, *Pericarpium Citri Reticulatae (Chenpi)*, *Rhizoma Atractylodis (Cangzhu)*, and *Radix Puerariae (Gegen)* (Zheng et al., 2022a). PWP has been utilized for many years in TCM for digestive diseases associated with dampness syndrome. Hesperidin, a flavanone glycoside found in citrus fruits, exhibits a wide range of pharmacological effects, including anti-inflammatory and antioxidant activities (Mahmoud, 2014; Sahu et al., 2013; Xu et al., 2009). Furthermore, previous research indicated that hesperidin may reduce experimental murine colitis (Xu et al., 2009). Glycyrrhizic acid, a bioactive triterpenoid saponin, acts as the principal active component of *Glycyrrhiza uralensis Fisch.* (Khan et al., 2013). Chen et al. demonstrated that glycyrrhizic acid mitigates myocardial ischemic injury by suppressing inflammation and oxidative stress (Xu et al., 2018). Luo et al. analyzed the chemical components of *Magnolia officinalis*, revealing its pharmacological effects on the digestive, nervous, cardiovascular, and cerebrovascular systems. Furthermore, it exhibits various properties, including antibacterial, anti-tumor, analgesic, anti-inflammatory, and antioxidant effects (Luo et al., 2019). *Atractylodes* exerts anti-inflammatory activity in gastric ulcer rats by down-regulating NF-κB and IL-1β while up-regulating IL-10 and IκBα. It corrects metabolic disorders associated with gastric ulcers, providing gastroprotective effects and effectively alleviating mucosal inflammatory injur*y* (Zhen et al., 2023). In this study, we found that treatment with PWP significantly downregulated the DAI in the model group, attenuated the levels of intestinal inflammatory factors IL-17, IL-6, IL-1β, and TNF-α, and upregulated the levels of tight junction proteins ZO1 and Occludin, thereby mitigating damage to the intestinal barrier. Combined with network pharmacology analysis to explore the pharmacodynamic mechanisms of PWP in the treatment of UC, the PI3K/AKT pathway may represent an important target for the action of PWP. Further validation through animal experiments revealed that PWP treatment significantly downregulated the expression levels of PI3K and the ratios of pAKT/AKT and phosphorylated pmTOR/mTOR in colonic tissues.

The regulation of gut microbiota and its metabolites may represent a key mechanism underlying the pharmacological effects of PWP. In the present study, model UC mice were prepared by combining high-fat diet combined with DSS exposure. Compared to the control group, mice in model group exhibited intestinal dysbiosis, characterized by an upregulation of opportunistic pathogens such as Staphylococcus, *Ruminococcus* torques group, *Desulfovibrio*, *Enterococcus*, and *Clostridium sensu stricto 1*. Notably, *Akkermansia*, a genus frequently recognized as a probiotic capable of alleviating intestinal inflammation, was significantly enriched in the feces of the model group. However, emerging evidence suggests that *Akkermansia’s* role in UC is not unequivocally beneficial. Excessive *Akkermansia* can deplete mucin in the gut, thereby compromising the mucus barrier and exacerbating intestinal damage (Qu et al., 2021; Yuan et al., 2022). PWP treatment significantly upregulated the levels of SCFAs-producing bacteria in the intestine, particularly *Alistipes* and *Parabacteroides*. This finding was further supported by GC-MS quantification, which indicated that butyric acid, valeric acid, and isovaleric acid were all significantly upregulated following PWP treatment. The gutMgene database was searched for SCFAs-related targets, and composite analyses were performed with targets related to the pharmacodynamic pathways of UC-associated PI3K/AKT/mTOR and autophagy associated with PWP. A total of ten composite targets were identified, among which butyric acid emerged as the key metabolite. In IBD, depletion of butyrate-producing bacteria can lead to loss of mitochondrial function and barrier integrity in intestinal epithelial cells, thereby triggering or exacerbating inflammation (Hamed et al., 2023). A recent study demonstrated that butyrate inhibits AKT phosphorylation via a PTEN-dependent pathway (Kimura et al., 2013). Similarly, in the present study, we found that PWP significantly reduced phosphorylation of proteins in the PI3K/AKT/mTOR signaling pathway in colonic epithelial cells. Fecal microbiota transplantation and butyrate supplementation experiments further confirmed that the gut microbiota and its metabolite butyrate are critical mediators in the PWP-induced inhibition of PI3K/AKT/mTOR pathway protein phosphorylation.

Autophagy plays a pivotal role in mitigating colonic inflammation in IBD. Selective autophagy of intestinal bacterial components (xenophagy) facilitates the degradation of luminal antigens that breach the mucosal barrier, thereby limiting colonic inflammation (Sharma et al., 2018). mTOR signaling, a key regulator of autophagy, suppresses autophagy by phosphorylating Ulk1 at Ser757 (Kim et al., 2011). In this study, PWP treatment increased LAMP1 expression, elevated the LC3B-II/I ratio, and promoted the consumption of the autophagy substrate P62 in colonic tissues, indicating enhanced autophagic activity. This upregulation of autophagy may contribute to the clearance of luminal antigens, such as lipopolysaccharide (LPS), that translocate following barrier disruption. Furthermore, butyrate, as an essential energy substrate for colonocytes, has been reported to restore energy metabolism and autophagy levels in germ-free mice (Donohoe et al., 2011). By elevating intestinal butyrate levels, PWP may restore colonic energy metabolism and sustain autophagic processes.

## 5 Conclusion

A mouse model was established through a high-fat diet combined with DSS. Mice in the model group exhibited significant intestinal barrier damage, activation of the PI3K/AKT/mTOR pathway, as well as disturbances in gut microbiota and abnormalities in the metabolism of SCFAs. Network pharmacological analysis indicated that PWP inhibited the activation of the PI3K/AKT pathway in UC. Experimental validation demonstrated that PWP treatment resulted in a significant downregulation of PI3K/AKT/mTOR pathway-related proteins and a concomitant upregulation of autophagy levels in colonic tissues, thereby mitigating inflammatory damage in the gut. Further gut microbiota co-metabolite analyses suggested that this may occur through the upregulation of colonic SCFAs-producing bacteria and the upregulation of intestinal levels of butyric acid, isovaleric acid, and valeric acid, which inhibit the activation of the PI3K/AKT/mTOR pathway and promote autophagy in colonic tissues. The role of gut microbiota and its metabolite butyrate was verified in subsequent FMT and butyrate supplementation experiments.

## Data Availability

The raw data supporting the conclusions of this article will be made available by the authors, without undue reservation.

## Supplementary material

Supplementary Table 1 Primers for qRT-PCR.

Supplementary Table 2 Compounds identified in the extract of Pingwei Powder.

Supplementary Table 3 Primary Antibody Information for Western Blot and Immunofluorescence.
